# Postless Hip Arthroscopy: A Technical Note on Obtaining Distraction

**DOI:** 10.1016/j.eats.2023.09.008

**Published:** 2023-12-25

**Authors:** Nels Leafblad, Josh Mizels, Travis Maak

**Affiliations:** Department of Orthopaedic Surgery, University of Utah, Salt Lake City, Utah, U.S.A.

## Abstract

Hip arthroscopy techniques continue to evolve. Historically, a post has been used as an aid to establish adequate hip joint distraction. However, this technique is associated with potentially devastating postoperative pudendal nerve injury and other urologic and gynecologic complications. In this article, we present our technique for postless hip arthroscopy.

The field of hip arthroscopy continues to evolve. One of its major recent advances has been the use of postless methods of hip distraction. Post-related complications include pudendal nerve injury and compression-related genitourinary and gynecologic soft-tissue injuries. Overall, post-related complications have been reported to be as high as 15% to 20%.^1^^,^^2^ In particular, the incidence of pudendal nerve injury reported in the literature ranges widely, from approximately 2% to 27%,^3^ likely owing to differences between prospectively and retrospectively collected data.^4^ Although most pudendal nerve injuries are indeed transient, resolving within weeks to months, in rare instances patients are left with permanent nerve damage. Transient or not, these complications can be uncomfortable and even devastating, especially for the young, athletic, and sexually active patient populations. Thus, methods to reduce these traction and compression-related injuries are critical. Postless techniques theoretically reduce the incidence of these injuries to 0%. Limiting traction time and magnitude of traction during surgery also have been shown to decrease the incidence of these traction-related injuries.^5^ In this Technical Note, we present our preferred technique for postless hip distraction.

## Surgical Technique (With Video Illustration)

Operating room (OR) table preparation is critical when using postless traction. We use Smith & Nephew’s hip arthroscopy bed attachment, the Hip Positioning System with Active Heel Technology (Smith & Nephew, Andover, MA). The Pink Pad (Xodus Medical, Pittsburgh, PA) is fixed to the table by wrapping the Velcro straps around the table. The pad should be placed as distal as possible and the patient positioned as proximal as possible, thus maximizing patient contact with the pad. The patient is anesthetized on the gurney and then transferred to the OR bed with a drawsheet under the patient. The draw sheet is then removed. Please refer to Table 1 for the list of equipment required.

**Table 1:** Equipment Used in Our Postless Hip Arthroscopy Technique

Hip Positioning System with Active Heel Technology (Smith & Nephew, Andover, MA)
Pink Hip Kit (Xodus Medical, Pittsburgh, PA)
Large C-arm fluoroscopy
Spinal needle
Nitinol guide wire
Hip arthroscopy instrumentation per surgeon preference

**Fig 1:**
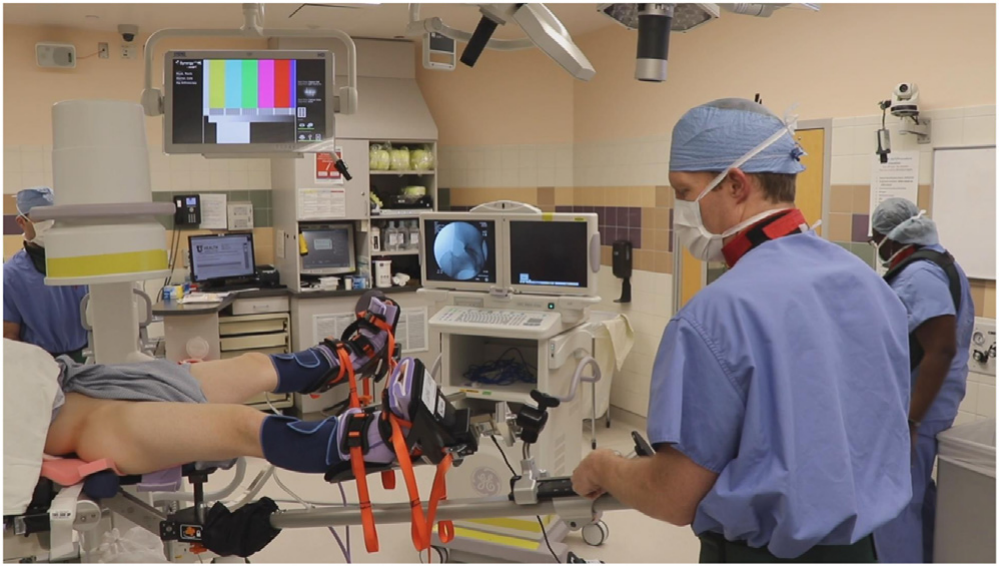
Operating room set up. C-arm comes in from side oppositive of the operative limb, which in this case is a right hip.

The patient is positioned so that the perineum is approximately 3 inches proximal to the end of the Pink Pad to allow for slight slippage during traction. The patient’s operative side arm is well-padded and placed across their chest. The patient is then secured to the bed with the safety strap positioned operative side arm and upper chest. The patient’s feet must be well padded and tightly secured in their respective boots. The C-arm is brought in from the nonoperative side of the bed. An anteroposterior (AP) image is taken so that the operative hip is centered on the image. The C-arm image should be adjusted as to match the preoperative radiograph of the pelvis. We typically leave the bed at 0° of Trendelenburg, although the table can be adjusted from 0° to 15° of Trendelenburg according to surgeon preference. We have found that 0° of Trendelenberg is all that is needed, even for lighter patients. Our preferred OR set-up is demonstrated in Figure 1.

**Fig 2:**
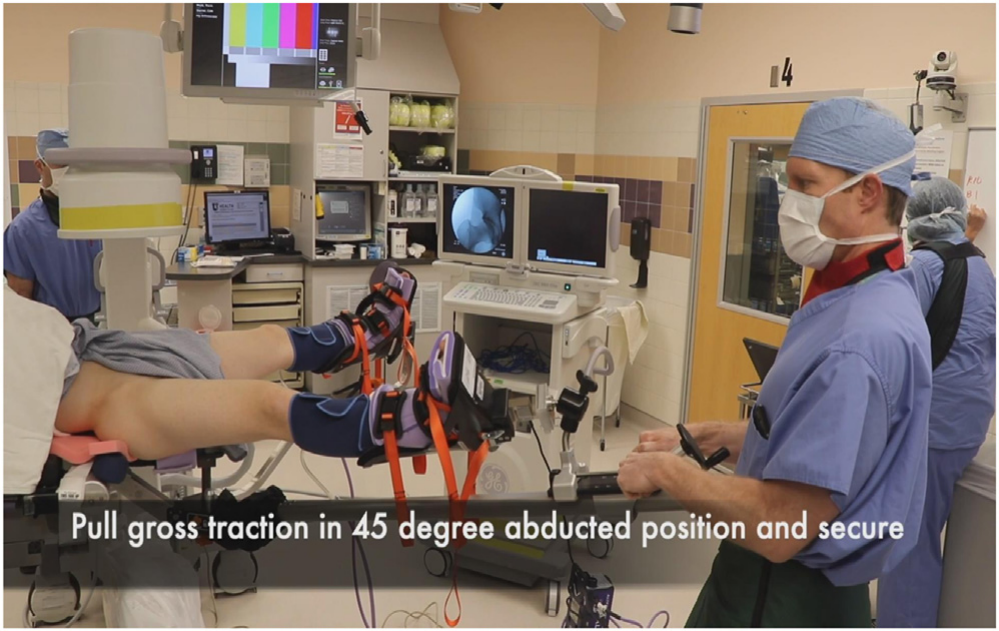
Pull gross in-line traction with operative leg in 45° of abduction and then secure/lock the gross traction (Right hip).

**Fig 3:**
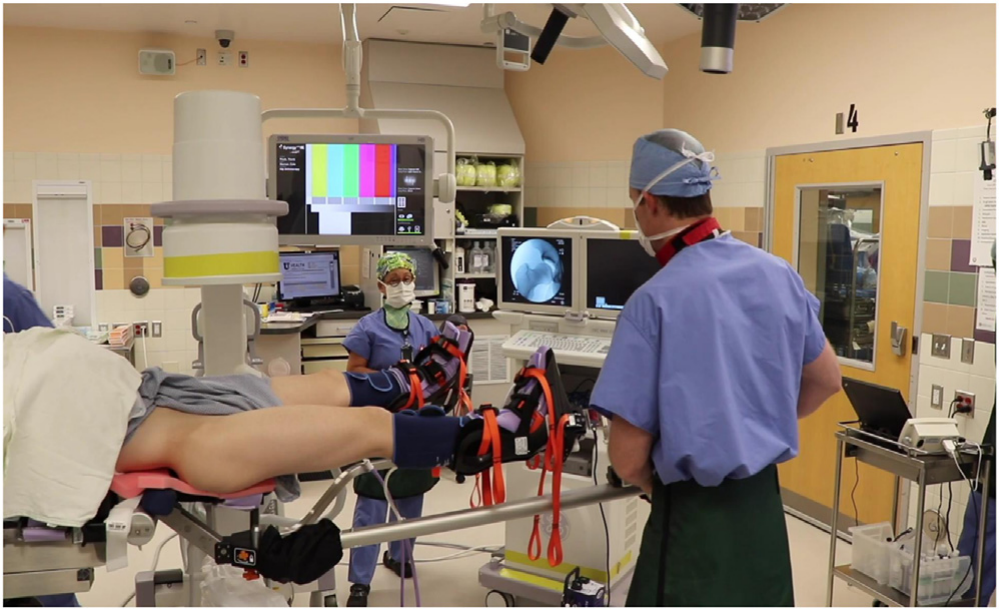
After pulling gross traction with leg in abduction, adduct the leg to neutral position with foot in slight internal rotation. Confirm adequate distraction on fluoroscopy (Right hip).

**Fig 4:**
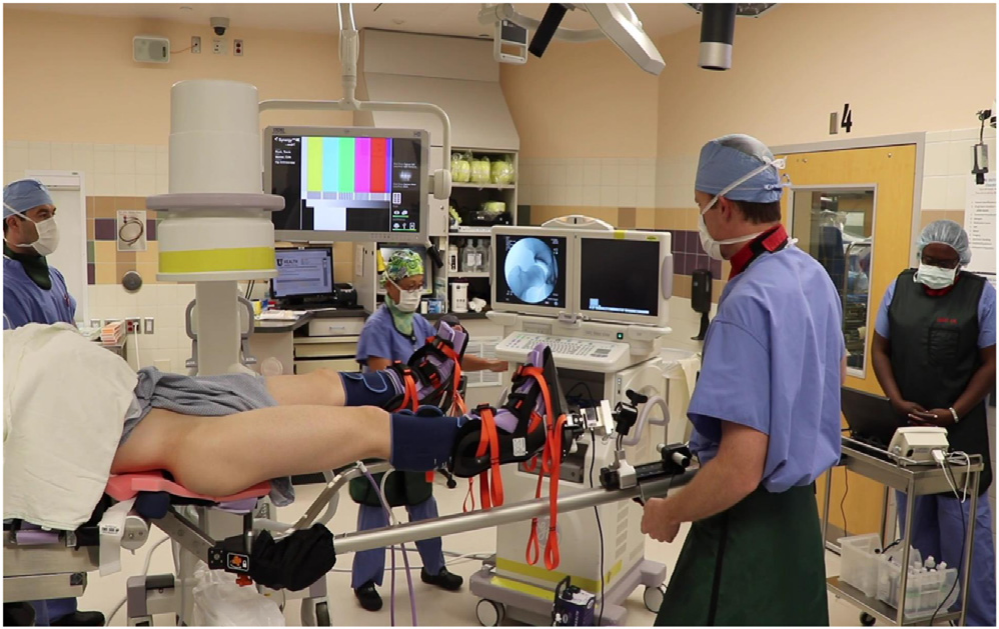
Apply fine traction as needed to obtain optimal hip distraction, confirming on fluoroscopy (Right hip).

At this time, gross traction can be applied to the hip. The operative leg is first abducted to 45° (Fig 2), locking the gross traction, and then immediately adducted to the neutral position. The foot is then rotated to neutral or slight internal rotation which typically breaks the suction seal of the hip joint. Fluoroscopy is used to confirm adequate distraction of the hip joint (Fig 3). If distraction from these maneuvers is less than desired, fine traction can be applied until the hip joint is appropriately distracted (Fig 4). Please refer to Table 2 for a summary of pearls and pitfalls.

**Table 2:** Pearls and Pitfalls

Pearls
Position the patient so that there is 3 inches of pink foam between the patient’s perineum and the end of the pink pad.
Securely strap the patient to the Pink Pad.
Securely place the patient’s feet in the boots.
Abduct operative leg to 45°, pull traction and lock the gross traction, then adduct the leg to neutral position, with neutral to slight internal rotation to break the suction seal.
If needed, one can vent the joint with a spinal needle and air arthrogram to break the suction seal if the above maneuver did not work.
Pitfalls
Placing the patient too far distal on the pink foam.
Not accounting for coxa profunda, in which case, a perineal post may provide better distraction.
Not placing lightweight patients in slight Trendelenberg position.

**Fig 5:**
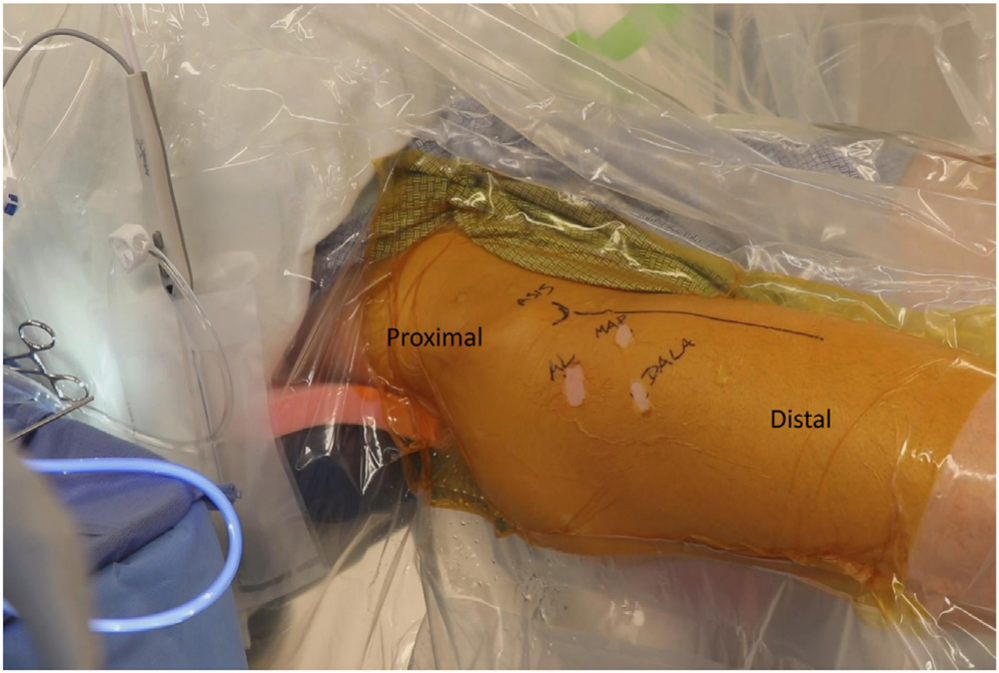
Marking out the anterior superior iliac spine (ASIS), anterolateral portal (AL), midanterior portal (MA), and distal anterolateral accessory portal (DALA) on a right hip.

**Fig 6:**
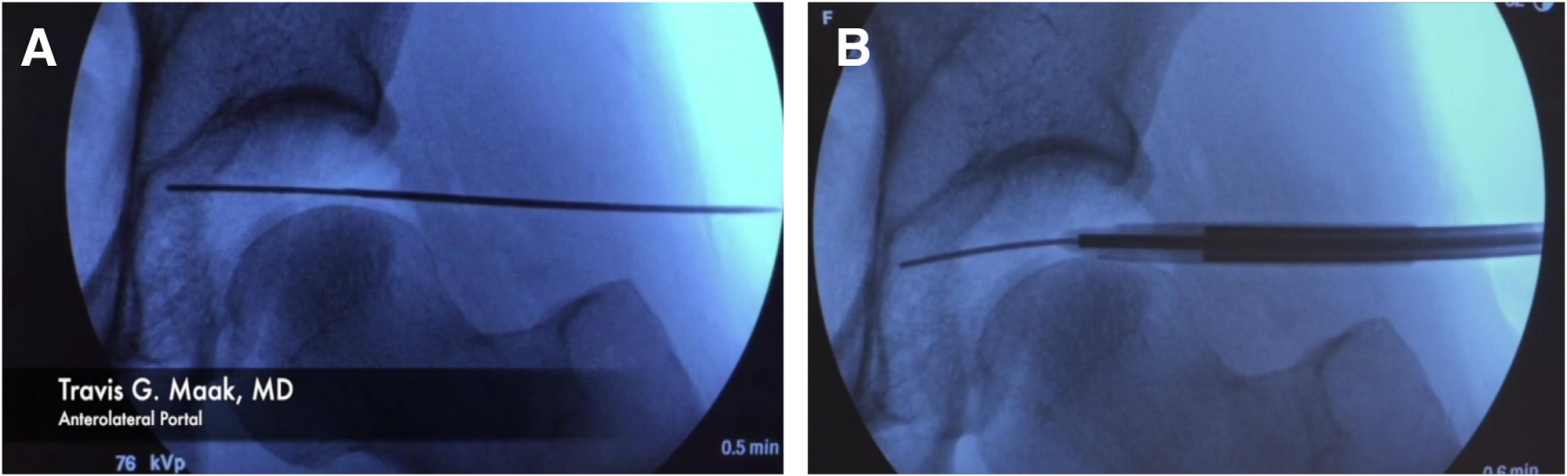
(A) Establishing the anterolateral portal by introducing a guidewire through the spinal needle. (B) Introducing the cannulated trocar over the guidewire under fluoroscopic control. This example is in a left hip.

After adequate hip distraction is obtained, the operative leg is prepped and draped in the preferred manner. Attention can then be turned to establishing the standard portals for arthroscopy. At our institution, an anterolateral (AL), midanterior (MA), and distal anterolateral (DALA) portals are most commonly used (Fig 5), but 2-portal arthroscopy is acceptable as well. First, we establish the AL portal by using blunt dissection, to allow for easy instrumentation, followed by a spinal needle. An air arthrogram can be used to further increase the amount of distraction without using more traction force (Fig 6A). The guidewire is then advanced followed by the trocar with cannula in an atraumatic fashion to avoid chondral injury (Fig 6B).

Next, the MA portal can be established like the AL portal using blunt dissection followed by a guidewire. The guidewire placement is typically confirmed arthroscopically, but can also be done under fluoroscopic guidance, and then the cannula is again placed atraumatically avoiding chondral or labral injury. The remainder of the arthroscopic hip surgery is then performed. View Video 1 to see the technique.

## Discussion

With this technique, we demonstrate that postless hip arthroscopy can be performed safely without compromising hip distraction or the efficiency of an operation. There are numerous benefits to this method, and we have found it easier to perform the operation without the post in place. We have not found the need to routinely perform percutaneous venting of the joint with a spinal needle in order to achieve adequate hip distraction. In addition, we have not needed to routinely place the patient in Trendelenberg position, as the friction created by the foam pad is generally all that is required. Lightweight patients may require slight Trendelenburg positioning, however. Although the authors have seldom found limitations to the postless method, there was a recent case report in which postless arthroscopy needed to be aborted in a patient with coxa profunda due to inadequate distraction. The patient later went on to have successful hip arthroscopy with a perineal post.^6^ Please refer to Table 3 for a list of advantages and disadvantages to postless arthroscopy. By sing postless traction, hip arthroscopists have the opportunity to eliminate gynecologic, urologic, and pudendal compression injuries caused by posts.

**Table 3:** Advantages and Disadvantages of Postless Distraction

Advantages
No risk of post-related perineal injuries
Less concern with traction time
Easy setup
Ease of obtaining distraction
Disadvantages
Occasional need for joint venting to obtain distraction
Potential for inadequate distraction in patients with coxa profunda

## Supplementary Data

ICMJE author disclosure forms

Video 1 We use Smith & Nephew’s hip arthroscopy bed attachment, the Hip Positioning System with Active Heel Technology (Smith & Nephew, Andover, MA). The Pink Pad (Xodus Medical, Pittsburgh, PA) is fixed to the table by wrapping the Velcro straps around the table. The pad should be placed as distal as possible and the patient positioned as proximal as possible, thus maximizing patient contact with the pad. The patient is positioned on the operating room bed so that the perineum is approximately 3 inches proximal to the end of the Pink Pad to allow for slight slippage during traction. The C-arm is brought in from the nonoperative side of the bed. An anteroposterior image is taken so that the operative hip is centered on the image. Gross traction is applied while the operative leg is abducted to 45° locking the gross traction, and then immediately adducted to the neutral position. The foot is then rotated to neutral or slight internal rotation. Fluoroscopy is used to confirm adequate distraction of the hip joint. If distraction from these maneuvers is less than desired, fine traction can be applied until the hip joint is appropriately distracted. Establish the anterolateral portal by using blunt dissection, followed by a spinal needle. An air arthrogram can be used to further increase the amount of distraction without using more traction force. The guidewire is then advanced followed by the trocar with cannula in an atraumatic fashion to avoid chondral injury. Next, the midanterior portal can be established using the same steps. The guidewire placement is typically confirmed arthroscopically and the trocar with cannula is again placed atraumatically avoiding chondral or labral injury. The remainder of the arthroscopic hip surgery is then performed.
